# Spatial-temporal distribution of debt and delinquency of the elderly in Thailand: Perspectives from the National Credit Bureau data

**DOI:** 10.1371/journal.pone.0306626

**Published:** 2024-07-08

**Authors:** Nij Tontisirin, Sutee Anantsuksomsri, Duangmanee Laovakul

**Affiliations:** 1 Faculty of Architecture and Planning, Thammasat University, Pathumthani, Thailand; 2 Regional, Urban & Built Environmental Analytics, Faculty of Architecture, Chulalongkorn University, Bangkok, Thailand; 3 Department of Urban and Regional Planning, Faculty of Architecture, Chulalongkorn University, Bangkok, Thailand; 4 Faculty of Economics, Thammasat University, Bangkok, Thailand; Universiti Teknologi MARA, MALAYSIA

## Abstract

Household debt in Thailand has become a serious problem, particularly for the elderly, because they have less ability to pay debts compared to the working population. Therefore, better understanding of the elderly’s debt and delinquency is crucial in policy formulation for the aged society. Previous works have focused on the elderly’s debt, either at the macro or individual levels. Little is known about geographic differences at the regional level. Knowing where the elderly debt and delinquency tend to cluster could guide area-specific policies to tackle the elderly financial stability. This research aims to examine the spatio-temporal distribution of the debt and delinquency of the near-elderly population (age 50–59) and the elderly (age 60 and above) using data from the National Credit Bureau of Thailand from 2008 to 2019. The analysis focuses on various types of loans. Spatial clusters are identified by Moran’s I and Local Geary statistics. The results show that the pattern of elderly debt and delinquency generally follows the life cycle hypothesis, except for credit card and business-related loans. There exist spatial clusters and spatial differences in different types of loans, suggesting regional heterogeneities that require area-specific policy responses.

## Introduction

The financial well-being of the elderly has become a pressing issue as the global demographic structure has shifted towards an aging society. The economic stability of the elderly is under intense scrutiny, with debt and delinquency emerging as significant challenges. Many elderly individuals rely on fixed incomes from social security, pensions, or retirement savings, which may not be sufficient to cover increasing costs of living, healthcare spending, and other unexpected expenses. This limited income makes managing debt more challenging and aligns with the life cycle hypothesis of saving [1, 2], which suggests that the elderly should not incur debts in the latter stage of their lives.

However, empirical evidence suggests otherwise. For instance, the elderly in the US still owe many debts, which tend to be carried on after retirement [3]. Having debts is also found to affect the elderly’s mental and physical health [4, 5], social loneliness [6], and financial security [3]. Delinquent debts can put even more pressure psychologically and physically on the elderly [7]. Empirical evidence of the elderly’s debts in the UK, US, and Europe is ample, but there has not been much study of this issue in Asia.

Being in debt and delinquent could be spatially dependent, which is influenced by geographic locations for many reasons. People living in neighboring locations may share similar socio-economic characteristics. For example, they may work in agricultural sectors and share similar debt patterns for agricultural tools. In rural areas where automobiles are the only available mode of transportation, people living there may borrow money to buy a private vehicle. In addition, people who live in natural disaster-prone regions may be in debt after being hit by an exogenous shock like a natural disaster. However, how debt and delinquency are geographically distributed is often underexamined. Previous studies have shown that spatial dependence was present in the operational efficiency of small and local cooperative banks [8] and in public expenditure of local governments [9], but very few examine the distribution of debt and delinquency, especially of the elderly.

Thailand serves as a distinct case study for the elderly’s debts. As a country in an emerging economy, Thailand’s household debts have recently increased dramatically [10]. The overall household debts in the first quarter of 2021 surged to a record high at 90.5 percent of the country’s GDP. Previous studies of loan-level data have shown that Thais tend to have debts at an early age, and the delinquent debt intensity increases with age and remains high after retirement [11, 12]. The rising debt and delinquency of the elderly have posed a greater challenge to the country’s financial stability since the elderly have reached 20 percent of the total population, making Thailand officially an aged society in 2022 [13].

Thailand is also a country with a high degree of uneven development. Bangkok, the capital of Thailand, and its vicinities in the central region have dominated the socio-economic development of the country. Other regions are poorer, more fragile, less diversified, and more reliant on agriculture, which is prone to changes in commodity prices and natural disasters [14]. These regional differences call for different analytical approaches and policy interventions. As Thailand has transitioned into an aged society, understanding the spatial and temporal patterns of elderly debt and delinquency is essential for policymakers, financial institutions, and social services aiming to enhance the financial security and overall well-being of older adults.

This research is guided by the following two main research questions: (1) to what extent is the intensity of elderly debt and delinquency varied across regions in Thailand? (2) where do we observe the high intensity of debt and delinquency of the elderly? It examines the geographic distribution of two indicators of debts and delinquency of the elderly, which are proxied by the average outstanding debt per borrower and delinquent debt per borrower, respectively. Specifically, the analysis compares the intensity of debts and delinquency of the near-elderly population (age 50–59) and the elderly (age 60 and above). The data are compiled from the loan accounts of the National Credit Bureau of Thailand from 2009 to 2018, aggregated at the postal code level, which is independent spatial units. Spatial autocorrelation methods are used to identify the geographic clustering of the intensity of debt and delinquency.

This research holds significant implications for understanding and addressing the issue of elderly debt and delinquency. By examining the spatial distribution and temporal trends of financial liabilities among the elderly, this study uncovered regional disparities and temporal changes that could underscore the need for tailored financial policies and support systems that can mitigate the risks and enhance the financial stability of older adults in Thailand.

## Spatial dependence of the elderly debt

Elderly debts have recently been one of the emerging issues in the financial health and well-being of the elderly. Many studies have been motivated by the life cycle hypothesis of saving, which focuses on the cyclical behavior of income-saving over a person’s lifetime [2]. The hypothesis suggests that, at an early part of their life, households must save in order to accumulate a stock of wealth to compensate for consumption when earning power dries up at the later part of their life. In other words, households go through saving in their early stage of life and through dissaving in the later part of their life. The life cycle hypothesis of saving has served as an underlying hypothesis of empirical studies of savings, income, and wealth on various scales, ranging from nations to households or individuals. As [1] puts it, “the hypothesis continues to provide the framework in which economists think about intertemporal issues at both the individual and economy-wide level.” Previous empirical studies have used the life cycle hypothesis as an analytical framework [15–20]. These analyses have direct policy implications on the social security system, savings for retirement, and economic growth as the global demographic shifts toward the aged society.

Empirical evidence from the US shows that desired debt follows the life cycle pattern [21]. Chantarat et al. [11] also found debt holding of Thai households followed the inverse U-shape pattern of the life cycle hypothesis, using loan account data from the National Credit Bureau of Thailand. Yilmazer and DeVaney [22] examine the likelihood of holding debt and the amount of debt over the life cycle for different types of household debt, using the 2001 Survey of Consumer Finances of the US. The results show that both the likelihood and the amount of debt decrease with age, supporting the implications of the life cycle hypothesis [22]. The analysis also found that poor, older households tend to have a harder time paying off their credit card debt. Poor households with heads older than 70 years tend to have a higher ratio of balances to total assets than households with a head age between 60 and 69.

Existing literature on household debt also suggests that there are repercussions of having debt among the elderly. Hiilamo [3] examines the interrelation between debt and the mental well-being of the elderly. Having a high debt-to-wealth ratio is positively correlated with lower mental well-being. Worrying about debt burden is also positively correlated with feelings of social loneliness among the elderly [6]. Carrying debt burdens is also found to affect both the physical and mental health outcomes of the elderly in the US [5]. Carrying debt through retirement affects retirees’ financial security as they have more expenses to cover with limited resources. Indebted older households have incentives to delay retirement or change the timing of their Social Security claim to avoid the inability to repay their debt [3]. Financial security in retirement can be greatly influenced not only by a household’s level of savings and assets but also by the amount of debt accumulated [23]. People will either retire later or work to generate some income during retirement to save more.

Although there are extensive studies on the socioeconomic effects of elderly debt, very few studies have focused on its spatial dimensions to identify where high debt and delinquency cluster. In fact, spatial dependence could be observed in elderly debt. For example, elderly debt may be spatially dependent, with higher levels of debt in areas with higher costs of living. Understanding geographic differences in debt and delinquency, especially among the elderly, could reveal the financial security of the elderly in specific areas, which could guide area-specific policy responses to help uplift the livelihood of the elderly. Therefore, this study contributes to the current literature by focusing on the spatial and temporal distribution of the debt and delinquency intensity of the elderly, using Thailand as a case study.

## Regional characteristics in Thailand

According to the National Geographic Committee of Thailand, there are six regions in Thailand: Central, East, North, Northeast, South, and West. These regions are defined according to their socio-economic and geographic similarities [24]. Table 1 shows the total population in 2021, percent share of the urban population, percent share of the elderly population (age 60 and above), total land area, number of provinces, Gross Regional Product (GRP), and GRP share by sector, agriculture (agr), manufacturing (man), and service (ser) sectors, of each region in Thailand. The urban population indicates the number of people living in municipalities as defined by the National Statistical Office of Thailand.

**Table 1:** Regional characteristics.

Region	Total Population (2021)	Urban Pop (%, 2021)	Elderly Pop (%, 2021)	Land Area (%)	Gross Regional Product^a^ (Million THB, 2019)	Share of GRP (%, 2019)		
						Agr	Man	Ser
Central	20,175,194	52%	20%	18%	8,887,830	1%	26%	73%
East	4,963,414	46%	16%	7%	3,042,916	6%	64%	29%
North	6,320,651	32%	26%	18%	1,299,834	25%	20%	55%
Northeast	21,826,920	20%	17%	33%	1,596,094	19%	22%	59%
South	9,454,193	28%	16%	14%	1,473,623	22%	15%	63%
West	3,474,964	28%	24%	10%	597,788	20%	32%	48%
**Total**	**66,171,439**	**34%**	**19%**	**100%**	**16,898,086**	**8%**	**31%**	**61%**

Author’s compilation from the Department of Provincial Administration (DOPA) & Office of the National Economic and Social Development Council (NESDC).

^a^Regional definitions of the NESDC are slightly different from the National Geographic Committee of Thailand. Gross Regional Product is from the NESDC.

As can be seen in Table 1, regional heterogeneities are prominent in Thailand. Urbanization is related to regional economic development. Highly urbanized regions have higher economic levels of economic outputs, measured by GRP. The Central region—where the capital city of Bangkok is located—is the most urbanized, with over half of its population living in municipalities. Its economy is predominantly service-oriented at 73 percent of the GRP, compared to the national average. The Eastern region is the second highest in terms of urbanization and GRP, with its strong manufacturing base. The Northeast region is the largest and most populated but the least urbanized, a significant rural economy with high agricultural and lower manufacturing shares in comparison with the nation. The North, South, and West share some similarities in terms of percent urban population, with around one-third of the population living in municipalities. The South’s service sector is slightly above the national average due to its coastal tourist attractions. In 2021, the elderly population accounted for 19 percent of the total population, with the highest percent share in the North and the lowest in the South and in the East.

**Fig 1:**
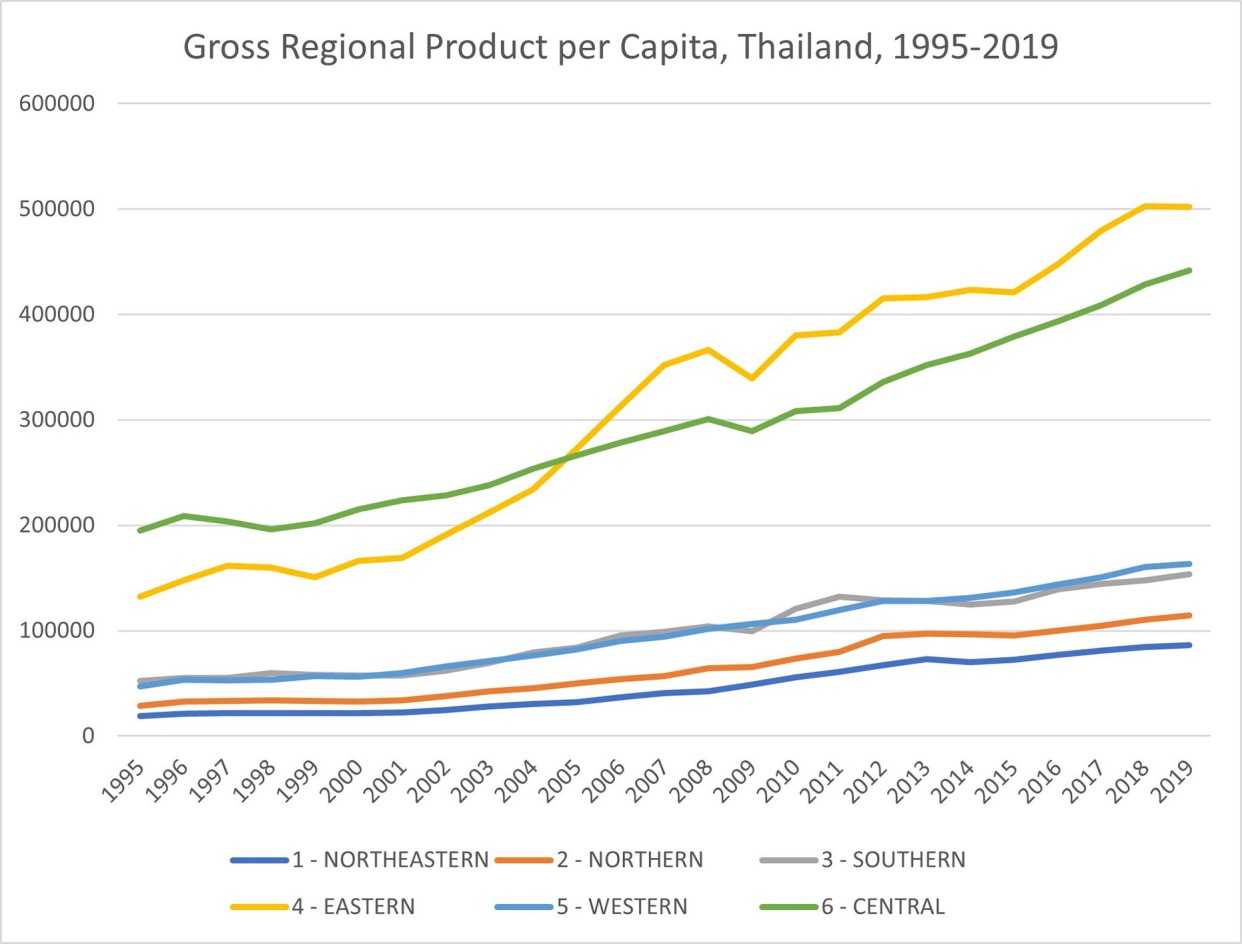
Gross regional product per capita, Thailand, 1995–2019. From Office of the National Economic and Social Development Council (NESDC).

Regional developments in Thailand, as measured by the GRP per capita, are also uneven over the year. Fig 1 shows diverging trends of the GRP per capita. The GRP per capita of the Central and Eastern regions are much higher than the rest of the regions. Several previous studies have shown that the robust economic growth of the Eastern region was a result of the Eastern Seaboard project, which promotes industrialization through foreign direct investments [25].

Studies on household debts in Thailand are quite sparse. Thais tend to incur debts from a young age, and these debts tend to be carried on after retirement [11]. Household debts are found to be correlated with financial health [26]; Thai households with mortgage tend to have better financial health than those without debts. On the other hand, households with consumption debts tend to have less financial health. The financial health of households is measured by the survival ratio, which is the ratio between total income and expenditure. Determinants of household debts include age, marital status, education, and region of residence. Households in the northeast and south regions were found to be less financially healthy than households in Bangkok. Yet, there are not many investigations of household debts that take into account spatial dimensions explicitly.

## Methodology and data

### Data

The main data of this analysis is drawn from two main data sets. The first is the Loan Account Data (LAD) from the **National Credit Bureau (NCB)**. Established in 2005 through the merger of Central Credit Information Services and Thai Credit Bureau companies, the NCB functions as a central hub for credit data collection, aggregating loan information from various financial institution members, including government financial entities, commercial banks, and other financial establishments. These financial institution members are required to share loan information to the NCB on a regular basis. The credit data collection of the NCB empowers its member institutions to enhance lending assessments, increase efficiency, and minimize the risk of nonperforming loans, ultimately contributing to the stabilization of the economic system [27].

The LAD used in this analysis is the consumer loan data aggregated at the postal code level, which consists of 959 postal areas. Thus, the unit of analysis is the postal code level. The postal code recorded in the LAD represents the lender’s mailing address, which may be a residential address or work address. The data range from 2009 to 2018, reported as of December 31^st^ of each year.

Table 2 describes the data from the NCB. Key variables include outstanding loan, delinquent debts, the number of accounts, and the number of account holders. The outstanding loan is the loan balance expected to be paid by the borrower. The Delinquent debts are the loan amount past due for more than 90 days, as defined by the NCB. The number of accounts is the total number of account at a particular postal code, and the number of account holders is the total number of people who have loan accounts. One person may have many different loan accounts; for example, an automobile loan and a credit card loan constitute two loan accounts for one lender. Thus, to represent the amount of delinquent loans per person, average delinquent loans per borrower was selected as a key variable as opposed to average loans per account.

**Table 2:** Loan account data description.

Variable	Description	
Postal code	Five-digit postal code of Thailand	
Province	Province in Thailand (77 provinces)	
Region	Six regions: Central, East, North, Northeast, South, and West	
Year	2009–2018 (data as of December 31^st^ of each year)	
Outstanding loan	Aggregated amount of outstanding loan balance (Thai Baht)	Available by loan type and by age group. **Loan type:** • Total loan • Housing loan • Automobile loan • Credit card • Personal loan • Other loan (business-related loan) **Age group:** • All ages • 50–59 years old • 60 years and older
Delinquent debt	Aggregated loan amount past due for over 90 days (Thai Baht)	
Number of the account	Aggregated number of account holders	
Number of the account holder	Aggregated number of account holders	

The data are reported in six account types: total loans, housing loans, automobile loans, credit card loans, personal loans, and others. Automobile loans include both hire-purchase and leasing of automobiles and motorcycles. Other loans are business-related loans, including hire-purchase for agriculture, nano-finance, and commercial loans. The data are also reported by age group: all ages, the near-retirement or pre-retiree group (age 50–59) and the retiree group (age 60 and above). Note that the retirement age in Thailand is 60 years old.

The second data set is postal code geographies in the geographic coordinate systems (GIS). There are 955 postal codes in Thailand. These postal areas can also be defined administratively by province and region. The postal areas cover an average of approximately 905 square kilometers, with a minimum of 3.9 square kilometers and a maximum of 48,409 square kilometers. The georeferencing of LAD was carried out using postal code geographies. The LAD lacking postal codes or containing foreign postal codes were excluded from the analysis.

### Analytical method

The analysis primarily consists of two components. It first explores regional differences in the intensity of debt and delinquency, which are measured by average debt and delinquent debt per borrower, respectively, by age group and by type of loan. The regional averages are weighed by the number of borrowers. For delinquent debt, the averages are weighted by the number of delinquent borrowers.

The second component of the analysis focuses on the spatial distribution of delinquent debt per borrower at the postal code level. The analysis of spatial clusters is performed using Geographic Data Analysis or GeoDa, which implements exploratory spatial data analysis for lattice data [28]. Moran’s I statistic is reported to show global spatial autocorrelation. Local clustering is illustrated by cluster maps.

Spatial autocorrelation is a method used to identify the correlation of average debts or delinquent debts per borrower with geographic location. The underlying assumption of spatial autocorrelation analysis is based on *Tobler’s First Law of Geography*, which indicates that "everything is related to everything else, but near things are more related than distant things" [29]. In this analysis, spatial autocorrelation is measured by Moran’s I statistic as follows:

$$I=\frac{n}{S_{0}}\frac{\sum_{i=1}^{n}\sum_{j=1}^{n}w_{i,j}z_{i}z_{j}}{\sum_{i=1}^{n}z_{i}^{2}}$$ (1)

Where *z_i_* denotes the mean deviation of aggregated loans of postal code *i*,

*w_i,j_* denotes the spatial weight between postal codes *i* and *j*,

*n* denotes the total number of postal codes,

*S*_0_ denotes the aggregate of all the spatial weights, such that $S_{0}=\sum_{i=1}^{n}\sum_{j=1}^{n}w_{i,j}$.

The value of Moran’s I statistic ranges from -1.0 to +1.0. A positive Moran’s I indicates a spatial cluster, either from high values clustering near high values or low values clustering near low values. On the other hand, a negative Moran’s I indicates that the distribution of high values tends to be near low values, and vice versa. After the Moran’s I statistic is computed, the Expected Moran’s I is permutationally computed and compared to the observed one. A statistical test is performed to determine whether the difference between the observed and expected statistics is statistically significant under the null assumption of features being randomly distributed across the study area. For detailed discussion, see [30].

To identify spatial clustering, the Getis-Ord G statistic is used [30, 31]. The statistic is a ratio of the values of neighboring locations to the total values. The *G* statistic is defined mathematically as follows:

$$G_{i}=\frac{\sum_{j\neq i}^{n}w_{i,j}x_{j}}{\sum_{j\neq i}^{n}x_{j}}$$ (2)

Where *x_i_* denotes the aggregated loans of postal code *i*

The *G* statistic can be interpreted as follows: if the statistic is greater than the mean, a high-high cluster or hot spot is identified; if the statistic is lower than the mean, a low-low cluster or cold spot is identified. The statistic is also tested for its significance with the null hypothesis of no spatial association. In practice, conditional random permutation is used for statistical inference [32].

As shown in previous equations, a spatial weight is an important component of the spatial autocorrelation analysis. A spatial weight characterizes spatial relations among features in the data [33]. It can be defined numerically in an N-by-N matrix of spatial relationships. Conceptually, spatial relationships can be specified in various ways, for example, inverse distance, contiguity, and space-time relations. Data in the Geographic Information Systems (GIS) can specify spatial relations based on their geographic features.

Properly defining spatial relationships is recognized as a difficult and controversial task [34]. Appropriate spatial relationships should reflect the properties of spatial processes which interplay among characteristics. Distance decay or inverse distance modeling of spatial relationships is often used to specify influences among features through proximities. The influence becomes smaller as features are farther apart. Contiguity spatial relationship is appropriate when data are polygons and similar in terms of size and distribution. K nearest neighbors spatial relationship is suitable when the analysis requires a minimum number of neighbors for each feature. In this analysis, we employ the inverse distance matrix to detect spatial connections between postal areas, as the proximity may influence the financial indebtedness and delinquency rates among the elderly.

## Analysis and discussion

### Descriptive statistics

**Fig 2:**
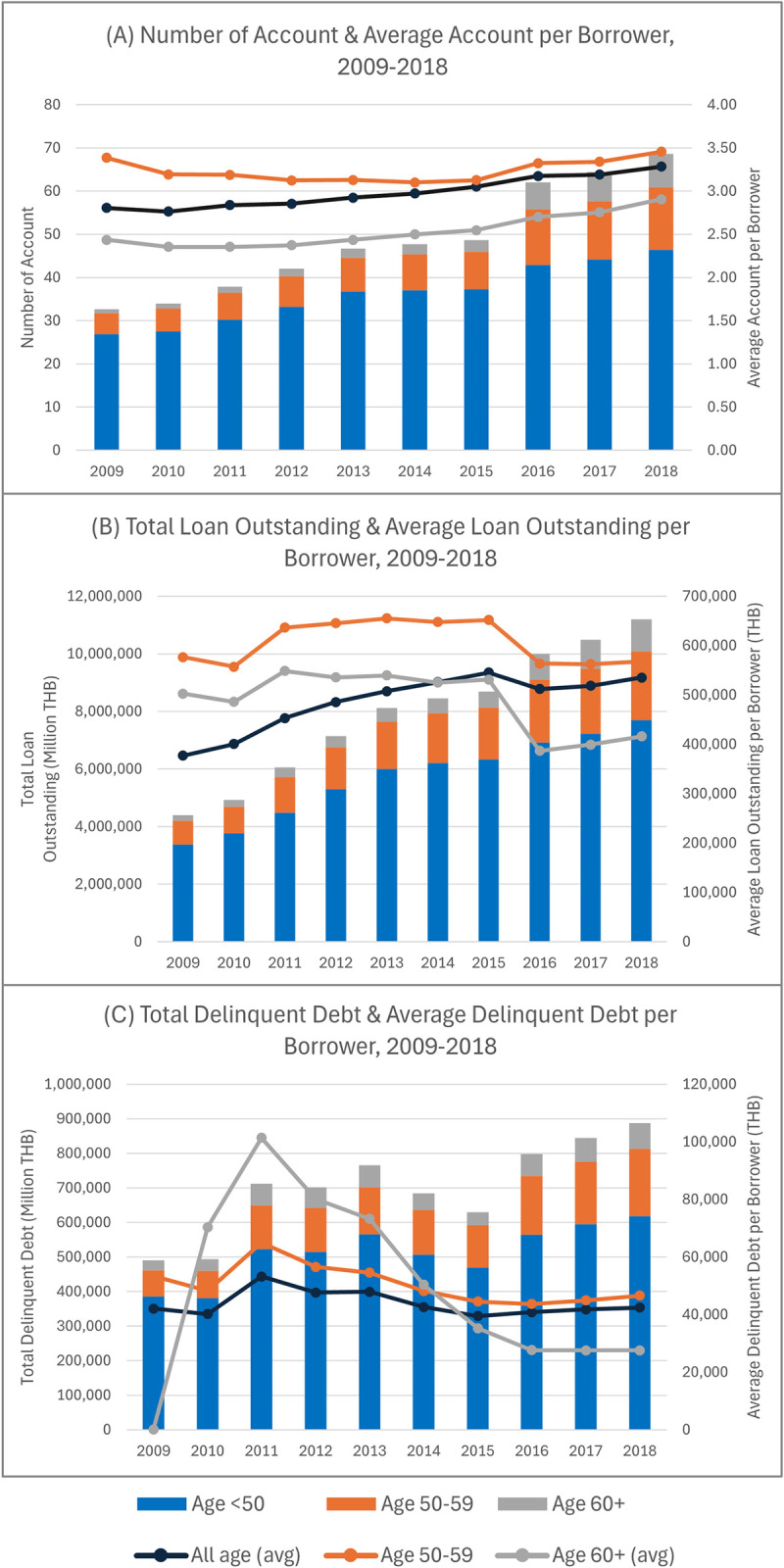
Number of account, total loan outstanding, and total delinquent debt by age group, 2009–2018.

The NCB data show that access to credit expanded considerably after 2009. The number of financial institutions grew from 73 in 2009 to 97 in 2018. Fig 2 illustrates three main indicators of access to credit by different age groups: (A) the total number of accounts and the average account per borrower, (B) the total loan outstanding and its average per borrower, and (C) the total delinquent debt and its average per borrower. In each graph, the lines represent the averages per borrower, displayed with the axis on the right.

The total number of loan accounts has steadily increased since 2009, as illustrated in Fig 2(A). On average, each borrower had about three loan accounts. The pre-retiree group (aged 50–59) had the highest average number of accounts per borrower, indicating a relatively stable financial status compared to other age groups. Meanwhile, the average number of accounts per borrower for the retiree group (aged 60 and over) was lower and stable, with a slight upward trend.

The total loan outstanding has grown considerably since 2009, as shown in Fig 2(B). On average, loan outstanding per borrower of all ages steadily increased over time. The pre-retirees’ and the retirees’ average loan outstanding slightly increased from 2011–2015 but decreased continuously afterward. The significant increase in the average outstanding loan of this group in 2011 may be attributed to the 2011 flood in Thailand, which resulted in significant financial losses to the economy.

As shown in Fig 2(C), total delinquent debts had an increasing trend but fluctuated over time. The average delinquent debt of all ages peaked in 2011, with the retiree group having the highest and significantly declining afterward. In 2018, the average delinquent debt per borrower in the pre-retiree group was the highest, followed by all age groups, with the retiree group having the lowest average delinquent debt.

In summary, the NCB data from 2009 to 2018 has shown that the retirees still incur debts and delinquency but at a much lower level than the pre-retirees. The trend of average delinquent debt per borrower of the retirees also declined after 2014.

### Outstanding debts of the elderly

**Fig 3:**
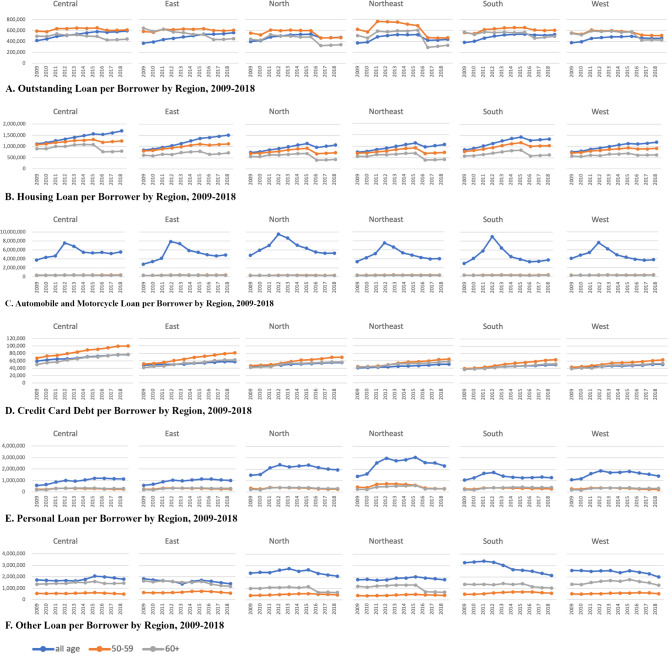
Outstanding debt per borrower by type of loan and by region, 2009–2018. Note: Types of loan include: (A) total outstanding loan, (B) housing loan, (C) automobile & motorcycle loan, (D) credit card, (E) personal loan, and (F) other loans. The data is represented in blue for all ages, orange for pre-retirees, and grey for retirees.

Fig 3 illustrates the average amount of outstanding loans per borrower for individuals of all ages, those nearing retirement, and retirees. The data is represented in blue, orange, and grey colors, respectively, across different regions from 2009 to 2018. The loans are categorized into six categories: (A) total outstanding loan, (B) housing loan, (C) automobile & motorcycle loan, (D) credit card, (E) personal loan, and (F) other loans. Regional averages are calculated using the number of delinquent borrowers as weights. Outstanding loan per borrower for all ages had an increasing trend in every region across the time horizon (Fig 3(A)). As suggested by the life cycle hypothesis, the average outstanding loans of the pre-retirees were higher than those of the retirees.

Outstanding amounts per borrower of these three groups also vary across different loan types, revealing diverse characteristics of borrowing. As for housing loans, it clearly follows the life cycle hypothesis that the retirees incurred the least amount of housing loans, on average (Fig 3(B)). Average housing loans of all ages were the highest, followed by those of the pre-retirees and the retirees, respectively.

As for auto loans, outstanding loan per borrower of all ages was also the highest, and actually much higher than those of the pre-retirees and the retirees (Fig 3(C)). The outstanding auto loan per borrower of all ages spiked in 2012, possibly due to the implementation of the first-car tax rebate policy, which provided a tax rebate for eco-car purchases by first-time buyers [35]. The policy was more likely to be applicable to younger car buyers who had never owned a car, so outstanding auto loans of all ages were much higher than for the other age groups.

On the other hand, outstanding credit card debt per borrower of the pre-retiree group was higher than both that of all ages and of the retiree group (Fig 3(D)). The outstanding credit card loans was also highest in the Central region. Like auto loans, personal loan per borrower of all ages were the highest across all regions (Fig 3(E)). The level of personal loans per borrower was particularly high for the Northeast and the North.

Other loans per borrower of the retirees were higher than the pre-retirees in all regions (Fig 3(F)). The level of loans per borrower of all ages was particularly high in the South, North, and West, while those of the retirees in the Central, North, South, and West were high.

### Delinquent debts of the elderly

**Fig 4:**
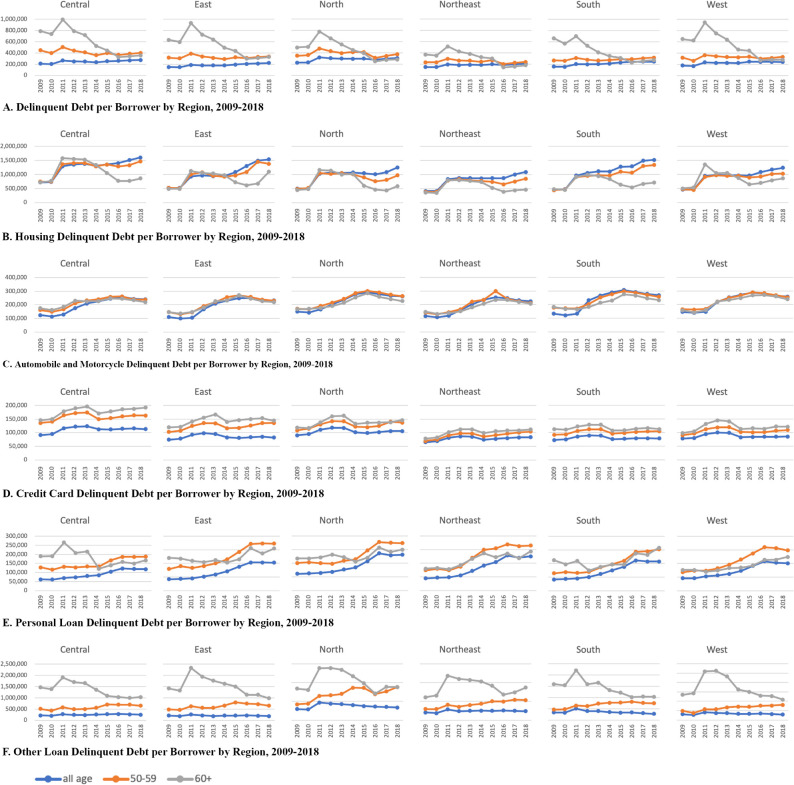
Delinquent debt per borrower by region, 2009–2018. Note: Types of loan include: (A) total outstanding loan, (B) housing loan, (C) automobile & motorcycle loan, (D) credit card, (E) personal loan, and (F) other loans. The data is represented in blue for all ages, orange for pre-retirees, and grey for retirees.

Fig 4 shows the regional differences in the average total delinquent debts per borrower of all ages, of the pre-retiree group, and of the retiree group from 2009–2018 by different types of loans. Regional averages are weighted by the number of delinquent borrowers. Overall, the levels of the average delinquent debt per borrower of all ages and of the pre-retiree group were quite stable throughout the period, while those of the retiree group reached their peak in 2011 and became smaller until the end of the period (Fig 4(A)).

Prior to 2016, the delinquent debt per borrower of the retirees exceeded that of the pre-retirees, which contradicts the life cycle hypothesis. The average total delinquent debt per borrower of the retirees in 2011 was the highest in the West and East, reaching almost 1.2 million THB per borrower. In every region, the discrepancies also reached their peak in 2011. One plausible explanation is that the 2011 great flood in Thailand might have led to incurred delinquent debts, especially for the elderly. However, the discrepancies between averages total delinquent debt per borrower of the pre-retiree group and that of the retiree group became narrower during this period, suggesting that delinquent debt per borrower of the retirees became smaller. Nevertheless, the level of the average delinquent debts of the retirees did not differ much from those of the pre-retiree group.

Average housing delinquent debt per borrower of the pre-retiree and retiree groups is somewhat similar across regions (Fig 4(B)). Overall, the average housing delinquent debts of the pre-retirees were stable after 2011, except for in the South where there was an upward trend. The averages of the pre-retirees in the Central and East regions were higher than in the rest of the regions, suggesting that housing prices in these two regions might be higher due to the higher level of urbanization. From 2009–2014, the average housing delinquent debts of the two groups were about the same level across all regions, except for those of the West, where the average of the retirees was much greater than that of the pre-retirees. Afterward, the average of the retiree group became less than those of all ages and the pre-retiree group in the Central, North, and Northeast regions, and these discrepancies widened after 2015. These patterns suggest that, although the elderly may still have housing delinquent debt, the level of these debts is not as much as the pre-retirees.

The level of the average automobile and motorcycle delinquent debt per borrower shared a similar pattern across regions for all ages, the pre-retiree group, and the retiree group. As shown in Fig 4(C), the average automobile delinquent debts of the three groups had an upward trend across the region, ranging between 100,000 to 200,000 THB in 2009, and between 200,000 to 300,000 THB in 2018. There was not much difference between the average automobile delinquent debts of the pre-retirees and the retirees. Nevertheless, these similarities between the pre-retirees and the retirees highlight the challenge of repaying delinquent debts for the retiree group since they tend to have less ability to pay off these debts than the pre-retiree group does.

Average credit card delinquent debt per borrower of the retiree group is alarming when compared to the pre-retiree group across the region (Fig 4(D)). Although the numbers only slightly increased from 2009 to 2018, the averages of the retirees were substantially higher than those of the pre-retirees throughout the period. Regional differences are apparent in terms of the level of the average delinquent debts, but all regions share common discrepancies between the retiree and the pre-retiree groups. The Central region is the region with the highest average credit card delinquent debts, reaching its peak in 2012 with almost 200,000 THB per borrower for the retiree group. Central, East, and North regions also share similar patterns, while the Northeast and the South have some similarities. Higher credit card delinquent debt per borrower of the retirees suggests that credit cards might be one of the main sources of financing after retirement. Borrowing with credit cards is easy. Repayments, for example, can be as small as the minimum payment, and the consequence of defaults on credit card loans does not involve immediately losing properties like other loans, i.e. housing or automobile loans. Nevertheless, the high level of delinquent credit card loans of the retirees reveals a challenge the elderly face in obtaining financial resources for consumption.

Personal loan delinquent debt per borrower for all ages and for the pre-retiree group share similar increasing patterns throughout the period, while those of the retiree group varied across regions (Fig 4(E)). Average personal loan delinquent debt of the retirees in the Central, East, North, and South was highest when compared to all ages and the pre-retirees during 2009–2013. Since 2014, the level of average delinquent debt of the retirees was lower than the pre-retirees, except for the South.

Of all the various types of loans, the average delinquent debt of other loans is most alarming for the retirees because the average delinquent debt of the retirees was much higher than the others (Fig 4(F)). Across all regions, the average delinquent debt spiked in 2011 and gradually declined since then. The level of delinquent debt per borrower of the retirees was also higher than that of other types of loans, averaging around one million THB. Since this type of loan includes business-related loans such as hire-purchase for agriculture, nano-finance, and commercial loans, high delinquent debt suggests the financial vulnerability of the elderly to this type of loan.

### Spatio-temporal cluster of delinquent debts

Previous analyses show regional differences in debt and delinquency of the retiree group compared to the all age and the pre-retiree groups. Understanding regional differences alone is not enough to create effective, targeted policies and allocate resources efficiently, including financial support and educational programs, for specific groups of elderly individuals. Identifying cluster structures in the delinquent debts can help pinpoint specific groups of elderly individuals who are more likely to experience debt and delinquency, enabling better monitoring and evaluation. This understanding facilitates targeted interventions and support systems tailored to the needs of these vulnerable populations.

In this section, the analysis specifically focuses on the spatial distribution of delinquency by various types of loan, by the analysis of the global Moran’s I by age groups, types of loan, and year (details shown in S1 Appendix). The statistics indicate significant spatial clusters across all types of loan and year for all age groups. For the pre-retiree group, spatial clusters are significant across the year for housing, personal loan, and other loan. For the retiree group, significant spatial clusters are found across the year for housing and credit card delinquent debts. Although there are some variations in the significance of Moran’s I, results strongly suggest that spatial clusters are present in the delinquency.

**Fig 5:**
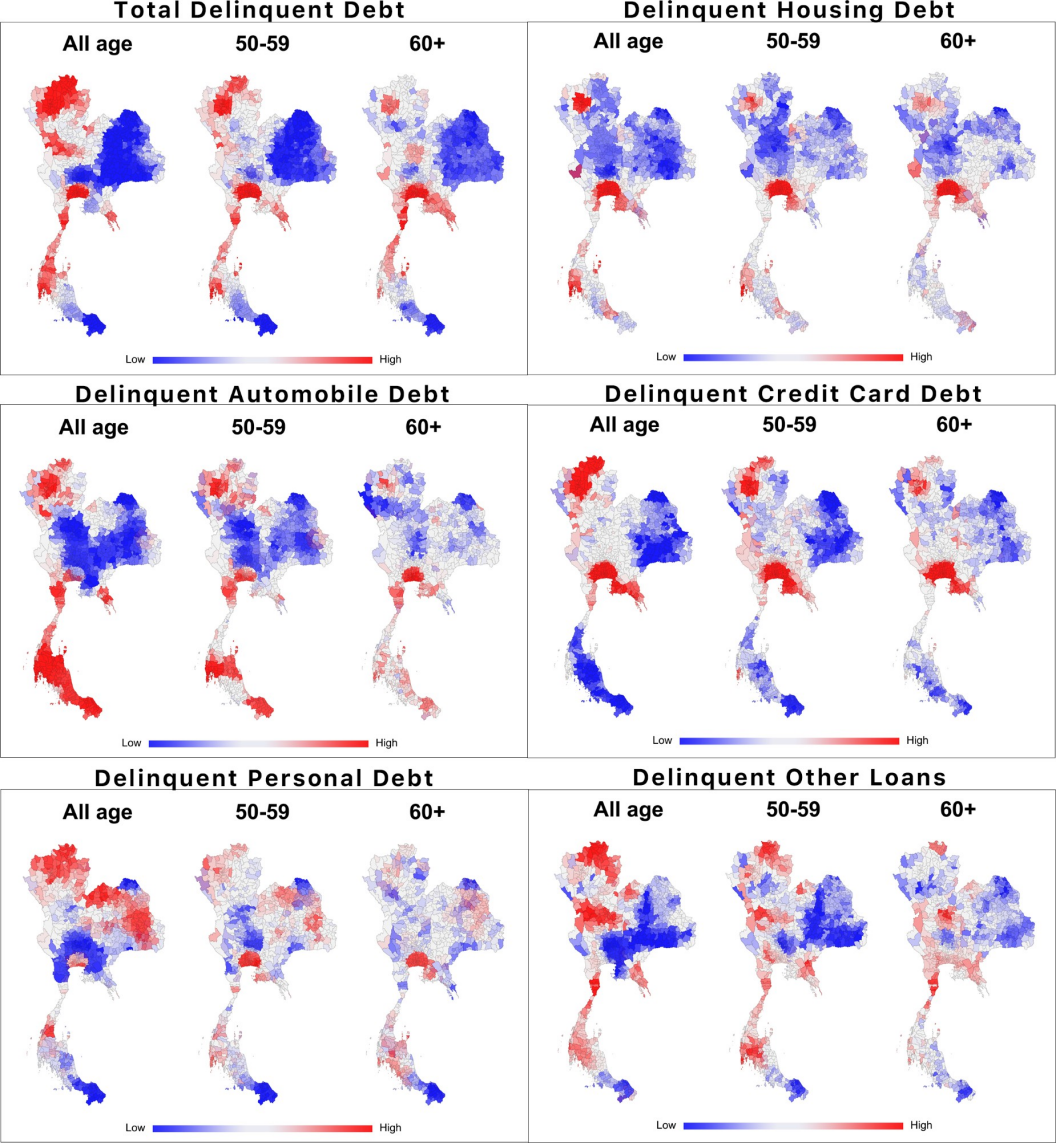
Spatial cluster of delinquent debt per borrower by type of loan and age group, 2009–2018.

Fig 5 shows the multi-year overlay of spatial clusters of the average delinquent debts per borrower by type of loan and age group from 2009 to 2018. Postal areas shown in red mean high spatial concentration of high levels of average delinquent debts, while areas shown in blue are low-level clusters.

As can be seen, spatial concentrations of each group can be observed in fewer areas as borrowers aged. For the retirees, spatial clusters of average delinquent debts are prominent in the Bangkok Metropolitan Region (BMR), East, West, and South regions. Like the retiree group, the pre-retiree group has similar clustering patterns with larger areas in the North. High average total delinquent debts per borrower of all ages are clustered in major urban areas like the BMR, Phuket, and Chiang Mai.

On the other hand, low-level clusters across these three groups are prominent in the Northeast and southernmost provinces. This concentration could mean people in these regions may not borrow as much, so they do not incur much delinquent debt. It can also be interpreted that people in these regions may not have the ability to borrow from lenders authorized by the NCB, i.e., due to their limited credentials, and may rely on unauthorized lenders such as non-bank or interpersonal loans. These unobservable phenomena may be beyond the scope of this current study but worth pursuing in the future.

As for housing loans, high-level spatial clusters also appear in the BMR, some provinces in the East, North, South, and West across all groups. Areas with high-level clusters tend to be highly urbanized, making the housing prices higher than in other areas. For example, Bangkok, Chon Buri, Chiang Mai, and Phuket have among the highest housing prices in Thailand. The high-level clusters are less prominent for the older population; only areas around the BMR and Chiang Mai remain highly clustered.

High-level clusters of average automobile and motorcycle delinquent debts are spread across the country, especially in the South, West, and North. These spatial patterns are reasonable because these areas do not have many transportation alternatives, so private vehicles like automobiles or motorcycles are the only option, especially in the South. High-level clusters of the retiree’s automobile delinquent debt remain in the BMR, the East and West close to the BMR, Chiang Mai, and the South.

Credit card delinquent debts are prominent in the BMR and its adjacent regions toward the East and West. Overall, high-level clusters of all the age group can be found around the BMR, the East, and the North. For the retiree group, spatial clusters are significant in fewer areas, and remain around the BMR and in the North around Chiang Mai.

Clusters of delinquent personal loan are significant in the BMR, the North, Northeast, and South for the all age group. However, for the retiree group, besides the BMR and the East, spatial clusters of delinquent personal loan are significant in the South.

Average delinquent debt of other loans concentrated in different areas of the country when compared to other types of loans. High-level clusters of all age groups are prominent in non-urban areas in the North, Central, West, and South. For the retiree group, the concentration is in the West and Central regions.

### Implications of the spatial distribution of the elderly debts

The loan account data from the NCB aggregated at the postal code level clearly shows that the pattern of elderly debts in Thailand generally follows the life-cycle hypothesis. Although the discrepancies in the delinquent intensity of the pre-retiree and the retiree groups get smaller from 2009 to 2018, the fact that the retiree group still owes debts and delinquent loans is alarming for the financial stability of the elderly. It is essential to educate individuals on financial literacy and planning from an early age to ensure strong long-term financial well-being.

The average delinquent debts of the elderly tend to cluster in specific locations over time, which poses significant challenges for both policymakers and analysts. The analysis of this data requires the use of spatial statistical techniques that consider the spatial distribution of the data, as traditional statistical methods are not suitable for such data due to the presence of spatial autocorrelation. Areas with high levels of average delinquent debts are highly concentrated geographically, rather than randomly distributed. Therefore, it is necessary to use appropriate analytical methods to gain a better understanding of the challenges associated with the elderly’s debts.

The study’s findings indicate that there are regional variations in the distribution of delinquent debts among the elderly. The areas with high urban populations have higher levels of delinquent debts in housing and credit cards. Conversely, regions with dispersed urban areas have clusters of average delinquent automobile and motorcycle debts. These regional differences pose policy challenges, as there is no one-size-fits-all policy for the elderly’s debts. The financial needs of the elderly living in urban areas may differ from those in rural areas. For instance, pension schemes for individuals in rural areas may differ from their urban counterparts because they work in agriculture or informal sectors. Likewise, housing for the elderly in urban areas may differ from those in rural areas [36]. To develop effective policies, it is crucial to understand the socio-economic characteristics of the elderly who incur delinquent debts, along with geographic attributes.

The investigation into various types of delinquent debts among the elderly has revealed some important financial precautions for retirees. In particular, credit card delinquent debts of retirees are higher than those of pre-retirees across regions in Thailand. Although the level of delinquent debts is not as high as other types of loans, the fact that retirees have higher delinquent debts than pre-retirees is quite alarming. It strongly suggests that credit cards are being used as a source of financing for elderly individuals who may no longer have a consistent stream of income. These credit card debts tend to have high interest rates and can be prolonged with minimum payments, which can negatively impact the long-term financial health of the elderly. Geographically, those with credit card delinquent debts are predominantly in urban areas around the Bangkok Metropolitan Region (BMR) and the East, which are relatively more urbanized than the rest of the country. These findings from Thailand are consistent with previous research conducted in the US [15], indicating that credit card debts can be a critical financial issue for the elderly.

The lack of cash flow faced by many elderly individuals after retirement highlights the need for alternative sources of income. Additionally, the elderly tend to have lower housing loan burdens compared to other age groups, indicating that property ownership could be a significant asset for them. In light of this, reverse mortgages could be a viable option for generating cash flow after retirement [37]. A reverse mortgage is a type of loan in which borrowers use their homes as collateral, but instead of making payments to the bank, they receive regular payments from the bank. Although reverse mortgages were first introduced as a pilot project in Bangkok and Metropolitan in 2019 by the Government Housing Bank, they have not gained much popularity due to low participation rates. Therefore, it is essential to promote this option in other cities to provide financial security for the elderly population.

Spatio-temporal cluster maps also show the low intensity of delinquent debts in the Northeast and the southernmost provinces of Thailand for most types of loans. These results might be interpreted in two different ways. In the first interpretation, the elderly in these regions have no delinquent debts, and their financial health is decent. The second interpretive meaning of the results, however, may paint quite a different picture from the first. The elderly in these regions may have no loan because they may be unable to borrow from financial institutions due to no collateral or credit history. As such, the elderly in these regions have limited financial access. Further investigations are needed to explain these phenomena.

## Conclusion

This study examines regional differences and spatio-temporal distribution of debts and delinquency of the elderly in Thailand using loan account data from the National Credit Bureau from 2009 to 2018. The analysis compares the debts and delinquent debts of the pre-retiree population (age 50–59) and the elderly (age 60 and older) under the life-cycle hypothesis that the debts of the latter should be lower than the former. The debt intensity is measured by the average debt per borrower. The delinquent intensity is measured by average delinquent debts per delinquent borrower. The methods used to identify spatial clusters at the postal code level are Moran’s I for the global spatial autocorrelation and cluster maps of Geary’s c statistics for the local clustering.

The results have shown that the intensity of debt and delinquent debt is unevenly distributed across regions and across loan types. Spatial clusters are also present for the intensity of delinquent debt, suggesting that geographic distributions should be considered when analyzing debt and delinquency. The comparison between debt intensity of the retiree and pre-retiree groups shows that the debt intensity of the pre-retiree group is higher than that of the retiree group, as suggested by the life cycle hypothesis. However, the intensity of delinquent debt of the retiree group is higher than the pre-retiree group in some loan types, especially credit card and business-related loans. Geographically, credit card delinquency is highly clustered in urban areas, while business-related delinquent loan is more disperse.

The analysis of the elderly debt and delinquency under the framework of the life cycle hypothesis has revealed several implications for the economy and for policy responses. As the elderly may still incur some debts, extending the retirement age beyond 60 (or beyond 65 in other countries) may be a viable alternative for stabilizing streams of income for the elderly. As the demographic has shifted toward the aged society, jobs suitable for the elderly should be promoted in regions with high delinquent debts. In addition, financial literacy and plans for retirement should also be promoted from an early age.

## Limitations and further studies

Certain limitations are present in the study. First, since the data used in this study are drawn from the NCB of Thailand, the analysis is limited to debt and delinquency of loans only from its financial institution members such as government banks, commercial banks, and other financial institutions. The analysis does not include student loans, informal loans from non-financial institutions such as interpersonal loans, or loans from cooperatives. Secondly, the analysis conducted in this study was based on data aggregated at the postal code level, due to legal restrictions on privacy protection. As a result, individual socio-economic characteristics such as income, wealth, and demographic attributes were not included in the analysis. Previous studies have indicated that socioeconomic factors such as gender [26] could impact financial behaviors. However, due to data access limitations from the NCB, it was not possible to conduct the analysis at the individual level.

Third, the prevalence of the COVID-19 pandemic has shaped household debts, especially for rural households. The analysis was limited to the temporal availability of the data in the pre-COVID period. Nevertheless, it would be a baseline for further studies of the debt and delinquency of the elderly.

## Supporting information

S1 Appendix Global Moran’s I statistic of delinquent debt per borrower by year, 2009–2018. (DOCX)
