# Supplementary material for: Spatial-temporal distribution of debt and delinquency of the elderly in Thailand: Perspectives from the National Credit Bureau data
Source: PLoS One. 2024 Jul 8;19(7):e0306626. doi: 10.1371/journal.pone.0306626 (PMC11230550; doi:10.1371/journal.pone.0306626)
Supplement: S1 Appendix — (DOCX) [file pone.0306626.s001.docx]

S1 Appendix. Global Moran’s I statistic of delinquent debt per borrower by year, 2009-2018.

| **Variable** | **2009** | | **2010** | | **2011** | | **2012** | | **2013** | | **2014** | | **2015** | | **2016** | | **2017** | | **2018** | |
| --- | --- | --- | --- | --- | --- | --- | --- | --- | --- | --- | --- | --- | --- | --- | --- | --- | --- | --- | --- | --- |
| **Total Delinquent Loan** | | | |  |  |  |  |  |  |  |  |  |  |  |  |  |  |  |  |  |
| All age | 0.151 | *** | 0.154 | *** | 0.212 | *** | 0.315 | *** | 0.345 | *** | 0.305 | *** | 0.327 | *** | 0.387 | *** | 0.368 | *** | 0.345 | *** |
| 50-59 | 0.092 | *** | 0.12 | *** | 0.106 | *** | 0.15 | *** | 0.19 | *** | 0.285 | *** | 0.232 | *** | 0.407 | *** | 0.343 | *** | 0.289 | *** |
| 60+ | 0.072 | *** | 0.068 | *** | 0.098 | *** | 0.061 | *** | 0.061 | *** | 0.042 | *** | 0.064 | *** | 0.346 | *** | 0.278 | *** | 0.089 | *** |
| **Delinquent Housing Loan** | | | |  |  |  |  |  |  |  |  |  |  |  |  |  |  |  |  |  |
| All age | 0.218 | *** | 0.267 | *** | 0.196 | *** | 0.229 | *** | 0.293 | *** | 0.163 | *** | 0.264 | *** | 0.312 | *** | 0.339 | *** | 0.301 | *** |
| 50-59 | 0.098 | *** | 0.134 | *** | 0.119 | *** | 0.104 | *** | 0.149 | *** | 0.134 | *** | 0.073 | *** | 0.284 | *** | 0.214 | *** | 0.168 | *** |
| 60+ | 0.061 | *** | 0.068 | *** | 0.08 | *** | 0.081 | *** | 0.094 | *** | 0.131 | *** | 0.13 | *** | 0.148 | *** | 0.107 | *** | 0.093 | *** |
| **Delinquent Automobile Loan** | | | |  |  |  |  |  |  |  |  |  |  |  |  |  |  |  |  |  |
| All age | 0.22 | *** | 0.192 | *** | 0.208 | *** | 0.279 | *** | 0.325 | *** | 0.352 | *** | 0.242 | *** | 0.351 | *** | 0.356 | *** | 0.321 | *** |
| 50-59 | 0.01 |  | 0.04 | *** | 0.045 | *** | 0.08 | *** | 0.077 | *** | 0.17 | *** | 0.02 | *** | 0.179 | *** | 0.199 | *** | 0.2 | *** |
| 60+ | 0.02 | ** | 0.011 |  | 0.028 | *** | 0.016 |  | 0.035 | *** | 0.045 | *** | 0.059 | *** | 0.051 | *** | 0.053 | *** | 0.08 | *** |
| **Delinquent Credit Card Loan** | | | |  |  |  |  |  |  |  |  |  |  |  |  |  |  |  |  |  |
| All age | 0.261 | *** | 0.306 | *** | 0.297 | *** | 0.327 | *** | 0.332 | *** | 0.38 | *** | 0.344 | *** | 0.349 | *** | 0.342 | *** | 0.329 | *** |
| 50-59 | 0.121 | *** | 0.163 | *** | 0.169 | *** | 0.174 | *** | 0.254 | *** | 0.299 | *** | 0.222 | *** | 0.216 | *** | 0.195 | *** | 0.206 |  |
| 60+ | 0.075 | *** | 0.095 | *** | 0.116 | *** | 0.084 | *** | 0.117 | *** | 0.112 | *** | 0.099 | *** | 0.126 | *** | 0.126 | *** | 0.169 | *** |
| **Delinquent Personal Loan** | | | |  |  |  |  |  |  |  |  |  |  |  |  |  |  |  |  |  |
| All age | 0.21 | *** | 0.238 | *** | 0.206 | *** | 0.166 | *** | 0.175 | *** | 0.206 | *** | 0.202 | *** | 0.213 | *** | 0.206 | *** | 0.266 | *** |
| 50-59 | 0.108 | *** | 0.049 | *** | 0.066 | *** | 0.048 | *** | 0.109 | *** | 0.119 | *** | 0.086 | *** | 0.106 | *** | 0.127 | *** | 0.13 | *** |
| 60+ | 0.049 | *** | 0.045 | *** | 0.053 | *** | 0.026 |  | 0.024 |  | 0.036 | *** | 0.013 | * | 0.102 | *** | 0.079 | *** | 0.052 | *** |
| **Delinquent Other Loan** | | | |  |  |  |  |  |  |  |  |  |  |  |  |  |  |  |  |  |
| All age | 0.118 | *** | 0.056 | *** | 0.146 | *** | 0.194 | *** | 0.208 | *** | 0.141 | *** | 0.187 | *** | 0.219 | *** | 0.22 | *** | 0.185 | *** |
| 50-59 | 0.077 | *** | 0.054 | *** | 0.063 | *** | 0.06 | *** | 0.137 | *** | 0.124 | *** | 0.078 | *** | 0.136 | *** | 0.097 | *** | 0.093 | *** |
| 60+ | 0.07 | *** | 0.039 | *** | 0.056 | *** | 0.027 |  | 0.023 |  | 0.02 | ** | 0.035 | *** | 0.028 | *** | 0.034 | *** | 0.022 |  |

From the Authors’ calculations

Note: ***, **, * indicate Moran’s I statistic that is statistically significant at 0.01, 0.05, and 0.1 level, respectively.
